# A community based study on haemoglobinopathies and G6PD deficiency among particularly vulnerable tribal groups in hard-to-reach malaria endemic areas of Odisha, India: implications on malaria control

**DOI:** 10.1186/s12936-022-04358-5

**Published:** 2022-11-16

**Authors:** Sujata Dixit, Arundhuti Das, Ramakanta Rana, Hemant K. Khuntia, Akhil B. Ota, Sanghamitra Pati, Madhusmita Bal, Manoranjan Ranjit

**Affiliations:** 1 ICMR-Regional Medical Research Centre, Chandrasekharpur, Bhubaneswar, 751023 Odisha India; 2 School of Biotechnology, KIIT University, Bhubaneswar, 751024 Odisha India; 3 Scheduled Castes and Scheduled Tribes Research and Training Institute, Bhubaneswar, 751012 Odisha India

**Keywords:** PVTG, Malaria, G6PD, Sickle cell anaemia, Thalassaemia, Odisha

## Abstract

**Background:**

Haemoglobinopathies and G6PD deficiency are inherited disorders found mostly in malaria-endemic areas among different tribal groups of India. However, epidemiological data specific to Particularly Vulnerable Tribal Groups (PVTGs), important for planning and implementing malaria programmes, is limited. Therefore, the present community-based study aimed to assess the prevalence of haemoglobinopathies and G6PD deficiency among the 13 PVTGs found in the state of Odisha, reporting the maximum malaria cases in the country.

**Methods:**

This cross-sectional study was conducted from July 2018 to February 2019 in 12 districts, home to all 13 PVTGs, in an estimated sample size of 1461, selected two-stage sampling method. Detection of haemoglobinopathies was done by the variant analyser. Screening of G6PD deficiency was carried out using DPIP method followed by quantification using spectrophotometry. The PCR–RFLP technology was used to determine variant of G6PD deficiency and haplotype analysis of sickle cell, while ARMS-PCR and GAP-PCR was used for detecting the mutation pattern in β-thalassaemia and α-thalassaemia respectively. The diagnosis of malaria was done by Pf-PAN RDT as point of care, followed by nPCR for confirmation and *Plasmodium* species identification.

**Results:**

The prevalence of sickle cell heterozygotes (AS) was 3.4%, sickle cell homozygous (SS) 0.1%, β-thalassaemia heterozygotes 0.3%, HbS/β-thalassaemia compound heterozygote 0.07%, HbS-α-thalassaemia 2.1%, G6PD deficiency 3.2% and malaria 8.1%. Molecular characterization of β^S^ revealed the presence of Arab-Indian haplotype in all HbS cases and IVS 1–5 G → C mutation in all β-thalassaemia cases. In case of α-thal, αα/α-3.7 gene deletion was most frequent (38%), followed by αα/α-4.2 (18%) and α-3.7/α-3.7 (4%). The frequency of G6PD Orissa (131C → G) mutation was found to be 97.9% and G6PD Mediterranean (563C → T) 2.1%. Around 57.4% of G6PD deficient individuals and 16% of the AS were found to be malaria positive.

**Conclusion:**

The present study reveals wide spread prevalence of sickle cell anaemia, α-thalassaemia, G6PD deficiency and malaria in the studied population. Moderate to high prevalence of G6PD deficiency and malaria warrants G6PD testing before treating with primaquine (PQ) for radical cure of *Plasmodium vivax*. Screening and counselling for HbS is required for the PVTGs of Odisha.

## Background

Erythrocytic G6PD deficiency and haemoglobinopathies are the most common genetic disorders affecting millions of people worldwide [1]. These disorders, in their geographical distribution correlate remarkably with past and present endemicity of *Plasmodium falciparum* malaria [2, 3]. In India the G6PD deficiency and abnormal haemoglobins, such as haemoglobin S (HbS), haemoglobin E (HbE), haemoglobin D (HbD), β-thalassaemia (β^thal^) and α-thalassaemia (α^thal^) in varying proportions are predominantly prevalent among tribal communities [4–8] residing in central and north-eastern regions of the country known to be highly endemic for malaria [9]. The National Framework for Malaria Elimination (NFME) in India targets to eliminate malaria by 2030 and adopted the drug policy that stresses upon treatment of all confirmed malaria cases (Microscopy or Rapid Diagnostic Kit) with Chloroquine (CQ) for three days and primaquine (PQ) for 14 days in case of *Plasmodium vivax* and with ACT (Artesunate 3 days + Sulfadoxine-Pyrimethamine 1 day) accompanied by single dose PQ on day 2 in case of *P. falciparum* [10], without any specific policy to screen G6PD deficiency before administration of PQ. Despite the therapeutic advantages of PQ, the wider use of the drug is restricted by its toxicity profile in case of G6PD deficiency and the most important adverse effect of PQ is dose-related haemolysis, which could potentially create significant morbidity and undermine confidence in PQ prescription. Therefore, the diagnosis and management of G6PD deficiency are important for malaria control, which requires wider use of PQ for both reducing *P. falciparum* transmission and achieving the radical cure of *P. vivax* [11]. On the other hand, though heterozygotes for the sickle gene (Hb genotype AS) and α^thal^ are known to provide ~ 90% protection against death due to malaria [12–14] probably through accelerated clearance of *P. falciparum*-infected erythrocytes by macrophages, homozygous (Hb SS) SCD and α^thal^ patients are at increased risk of dying from malaria [13–15].

Odisha, an eastern Indian state with 4% of the total population contributes almost 40% of the annual malaria burden of the country, mostly reported from hilly forested areas inhabited by the tribal population, the majority of them are due to *P. falciparum* and *P. vivax* [5, 16]. Although separate studies have reported the prevalence of sickle cell, G6PD deficiency, and malaria in tribal-dominated areas of the state [17–21], but PVTGs-specific comprehensive data on these genetic disorders and their association with malaria are lacking, which is a pre-requisite for the institution of a cost-effective screening system as well as an intervention program. Earlier the Expert Committee on Tribal Health (2013) jointly constituted by the Ministry of Tribal Affairs and Ministry of Health and Family Welfare, Government of India and NITI Ayog, India (2018) have emphasised on generating information on important health issues such as sickle cell and malaria of these vulnerable population so as to achieve the UN Sustainable Development Goals (SDG) [22]. Realizing this, an attempt has been made here, to explore the burden of haemoglobinopathies and G6PD deficiency among the PVTGs residing in remote inaccessible areas with a high burden of malaria in Odisha.

## Methods

### Study areas and sample size

**Fig. 1:**
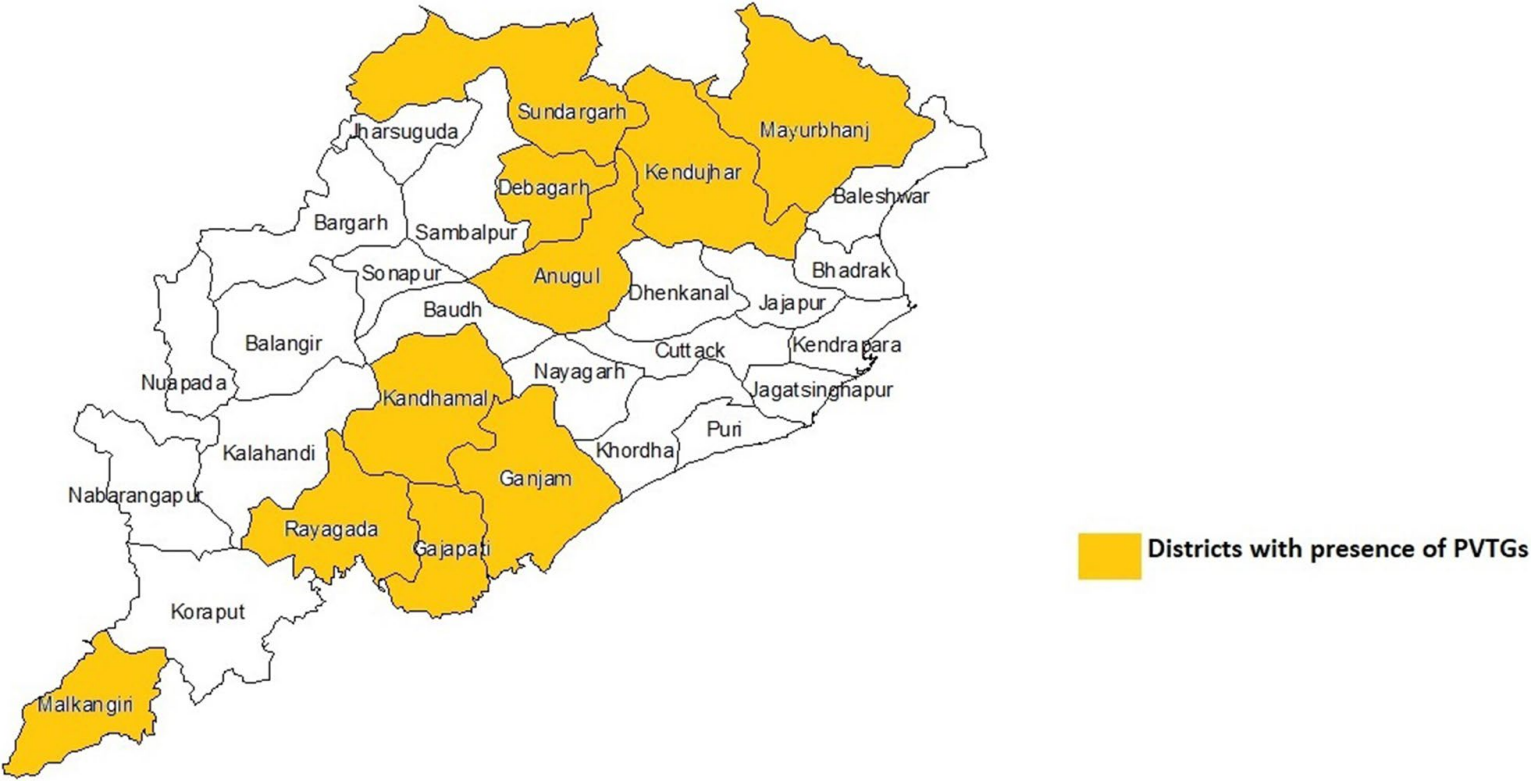
Map showing the study districts of Odisha state

A cross-sectional study was conducted from July 2018 to February 2019 among 13 PVTGs residing in 12 districts (Malkangiri, Kandhamal, Kalahandi, Rayagada, Keonjhar, Nuapada, Deogarh, Anugul, Sundargarh, Mayrubhanj, Gajapati, and Ganjam) of Odisha state. The sample size was estimated to 1461 individuals from 697 households using the formula, n = [DEFF*Np(1-p)]/[(d2/Z21-α/2*(N-1) + p*(1-p)] using OpenEpi software version 3.01. A two-stage sampling method was adopted to select the samples. Villages, the Primary Sampling Units (PSUs) were selected from a list of all the PVTGs villages from 12 districts with probability proportional to population size (PPS) sampling at the first stage followed by random selection of a sample of households within selected two PSUs from each PVTG at the second stage. The study was approved by the ethics committee of ICMR-Regional Medical Research Centre (RMRC), Bhubaneswar (Fig. 1).

### Laboratory investigations

At least 1 ml of venous blood was collected in BD Vacutainer^®^ EDTA tubes from each enrolled individual and transported within 24 h of collection in a cold chain to the ICMR-RMRC laboratory for further analysis. Complete blood cell count was done (CBC) by an automatic blood cell counter (Melet and Schloesing Laboratories, France) on the day of arrival.

### Screening and molecular analysis of haemoglobinopathy cases

Diagnosis of haemoglobinopathies was done by using cation-exchange high-performance liquid chromatography (HPLC; Bio-Rad VARIANT-II Haemoglobin Testing System) with the aid of the β-thalassaemia short programme.

For molecular analysis, the genomic DNA was extracted from 200 μl of whole blood using QIAGEN mini DNA extraction kit following the manufacturer’s instructions. DNA samples of sickle cell diseases and trait cases were screened for β-globin gene haplotypes (the HindII (5′ε), HindIII (Gγ), HindIII (Aγ), HindII (5′ψβ), HindII (3′ψβ), AvaII(β), and HinfI (β-3′) by restriction fragments length polymorphism and common α-globin gene deletions (–α3.7, –α4.2, –α20.5 and –MED, –SEA, –FIL, –THAI) by using published primers and methods [23, 24].

Five common mutations in the ß-globin gene including IVS 1–5 G → C, IVS 1 -1 G → T, Codon 41/42 (-TCTT), Codon 8/9, and the 619 bp in β-thalassaemia positive cases were carried out by ARMS-PCR technique using normal and mutation-specific primers [25].

### Screening and molecular analysis of Glucose-6-phosphate dehydrogenase deficiency

G6PD deficiency was detected by the Dichlorophenol-indophenol (DPIP) dye decolourization method and quantified by spectrophotometry following the method described by Dacie and Lewis [26]. The samples with enzyme activities < 2 IU/g Hb were considered as G6PD deficient in males and < 7 IU/g Hb were considered as G6PD deficient in the case of females [26]. Genotyping was done for the Orissa and Mediterranean variants only on DPIP-positive samples, using the PCR–RFLP method as described by Kaeda et al*.* [18]. At least 10% of all the experiments were repeated for quality control.

### Screening and species identification of malaria parasites

The Pf-PAN Rapid diagnostic kit (SD Biosensor, India) was used for the detection of *P. falciparum* and other *Plasmodium* infections using 20 μl of blood sample at the study site. Genomic DNA from all RDT positive samples was extracted from blood using QIAamp^®^ DNA Blood Mini Kit (QIAGEN, Germany) following the manufacturers’ manual. The DNA extracted was further subjected to species-specific nested PCRs as described by Snounou et al. [27]. The PCR was carried out in a Thermal cycler (Agilent, UK) and the PCR products, as well as RFLP sites/fragments, were subjected to 2.0% agarose gel electrophoresis followed by staining with 0.5% ethidium bromide. The species-specific bands (*P. falciparum*: 205 bp, *P. vivax*; 120 bp, *Plasmodium malariae*; 144 bp, *Plasmodium ovale*; 800 bp) were visualized and recorded digitally using the Gel-Doc system (Alpha Imager, USA).

### Statistical analysis

Data obtained were analysed and statistically tested using the software SPSS-20. Descriptive statistics were used to analyse the data obtained. The association test was done using one way-ANOVA (analysis of variance). The Hardy Weinberg Equilibrium (HWE) was tested using the Guo and Thompson method [28]. Chi-square test was performed to see if the population is in HWE.

## Results

A total number of 1461 individuals (male: 682, female: 779) with a mean age of 32.59 ± 20.92 years were included in the study. Among them 25% (n = 365) were children from 1 to 14 years, 48.9% (n = 715) were between 15 and 49 years of age and 26.0% (n = 381) were 50 years and above of age. In the case of sickle cell and thalassaemia, 57.1% were female and 42.9% were male and a majority of them were ≤ 50 years. Whereas in the case of G6PD 59.5% were male and 40.4% were female and 44.7% of the cases were between 15 and 49 years of age. G6PD and malaria were found to be more among males than in females (Table 1).

**Table 1:** Age-wise distribution of Sickle cell β-thalassaemia, G6PD deficiency, malaria cases among PVTGs

Disease condition	Total population	Male	Female	≥ 14 yr	15–49 yr	≤ 50 yr
Sickle cell + β-thalassemia	56	24 (42.9%)	32 (57.1%)	10 (17.9%)	22 (39.3%)	24 (42.9%)
G6PD deficiency	47	28 (59.5%)	19 (40.4%)	9 (19.2%)	21 (44.7%)	20 (42.6%)
Malaria positive	118	61 (51.7)	57 (48.3)	48 (40.7%)	49 (41.5%)	20 (16.9%)
Total	1461	682 (46.7%)	779 (53.3%)	365 (25.0%)	715 (48.9%)	381 (26.0%)

### Sickle cell and thalassaemia

Of the 1461 individuals screened, 3.4% (n = 50) were diagnosed as Sickle cell heterozygotes (AS), 0.1% (n = 2) Sickle cell homozygous (SS), 0.3% (n = 5) β-thalassaemia heterozygotes, 0.07% (n = 1) HbS/β-thalassaemia compound heterozygote and 2.1% (n = 30) α-thalassaemia. Of the 50 HbAS individuals, 30 (60%) were found to be associated (co-inheritance) with the α-thal gene. Amongst the α-thalassaemia individuals, 4% (n = 2) had double (α-3.7/α-3.7), 38% (n = 19) had αα/α-3.7 and 18% (n = 9) had αα/α-4.2 single gene deletion. A sequence haplotype analysis revealed the presence of Arab-Indian haplotype in all the HbS cases. Of the 13 tribes studied, β^S^ was found in eight tribes namely Juang, Kutia Kondho, Dongoria Kondho, Paudi Bhuyan, Langia Saora, Didayi, Bondo and Saora. While α-thal was found in five tribes i.e. Paudi Bhuyan, Dongoria Kondho, Langia Saora, Didayi and Saora (Table 2).

**Table 2:** Prevalence of Haemoglobinopathies and Malaria in Sickle cell anaemia among 13 PVTGs of Odisha

Name of PVTG	Total study population	Sickle-cell anaemia		α-thalassaemia + Sickle cell anaemia	β-thalassaemia	β-thalassaemia + Sickle cell anaemia	Malaria + Sickle cell anaemia
		Heterozygote (AS)	Homozygote (SS)				
Bonda	77	2 (2.6%)	ND	ND	ND	ND	ND
Chuktia Bhunjia	59	ND	ND	ND	ND	ND	ND
Didayi	84	6 (7.1%)	ND	3 (3.6%)	ND	ND	ND
Dongoria Kondha	145	3 (2.0%)	ND	2 (1.4%)	ND	ND	ND
Hill Kharia	48	ND	ND	ND	ND	ND	ND
Mankidia /Birhor	29	ND	ND	ND	ND	ND	ND
Juang	109	4 (3.6%)	ND	2 (1.8%)	ND	ND	ND
KutiaKondho	152	5 (3.3%)	ND	ND	ND	ND	4(80%) (Pf:4)
Lodha	102	ND	ND	ND	ND	ND	ND
LanjiaSaora	148	8 (5.4%)	ND	7 (4.7%)	ND	ND	ND
PaudiBhyan	309	10 (3.2%)	2 (0.6%)	8 (2.6%)	5 (1.6%)	1 (0.3%)	4(33.3%) (Pf:3,Pf + Pv:1)
Saora	199	10 (5.0%)	ND	8 (4.0%)	ND	ND	ND
Total	1461	48 (3.3%)	2 (0.1%)	30 (2.1%)	5 (0.3%)	1 (0.1%)	8 (16%)

*ND* not detected

Among all the haemoglobinopathy cases, 17.8% were children (≤ 14 years), 39.3% were in the reproductive age group (15–49 years) and 42.8% were above or equal to 50 years of age (Table 1). The β-thal gene was detected only in Paudi Bhuyan PVTG, where the DNA analysis showed IVS1-5 (G → C) mutation. The Hardy–Weinberg equilibrium (HWE) test showed that the Paudi Bhuyan population has not attained equilibrium for β^S^ and Saora for α^thal^ (Tables 3, 4).

**Table 3:** Phenotype distribution and allele frequency of Hb S, β-thalassaemia, α-thalassaemia among the unrelated 13 PVTGs of Odisha

Genotype	Unrelated PVTG
AA	1062
AS	40
SS	1
HbS (allele frequency)	0.019
AA	1103
Aβ	2
Hbβ (allele frequency)	0.0009
αα/αα	1081
αα/–α	23
–α/–α	1
Hbα (allele frequency)	0.0113

*AA* normal individual; *AS* sickle cell heterozygote; *SS* sickle cell homozygote; *HbS* sickle cell allele; *χ*^2^ chi square value; *αα/αα* normal α gene; *αα/–α* α gene single gene deletion; -*α/–α* α double gene deletion; *Hbα* allele frequency of α gene deletion; *Aβ* β gene mutation; *Hbβ* β allele frequency

**Table 4:** Tribe-wise phenotype distribution and allele frequency of HbS and α-thalassaemia among the unrelated 13 PVTGs of Odisha

PVTG Tribe	AA	AS	SS	HbS	*χ*^2^	αα/αα	αα/–α	–α/–α	Hbα	*χ*^2^
Juang	64	2	0	0.015	0.0156	ND	ND	ND		
Kutia Kondho	96	1	0	0.005	0.0026	ND	ND	ND		
Paudi Bhuyan	202	6	1	0.019	11.57*	205	6	0	0.0142	0.0439
Dongoria Kondho	115	2	0	0.009	0.008	115	2	0	0.0085	0.0087
Langia Saora	117	10	0	0.039	0.213	120	7	0	0.0276	0.102
Didayi	67	6	0	0.041	0.134	70	3	0	0.0205	0.0321
Saora	139	13	0	0.042	0.303	146	5	1	0.023	10.989*

*AA* normal individual; *AS* sickle cell heterozygote; *SS* sickle cell homozygote; *HbS* sickle cell allele; *χ*^2^ chi square value; *αα/αα* normal α gene; *αα/–α* α gene single gene deletion; *-α/–α* α double gene deletion; *Hbα* allele frequency of α gene deletion; *ND* not detected

^*^Statistically significant (p < 0.05)

### Glucose-6-phosphate dehydrogenase deficiency

**Fig. 2:**
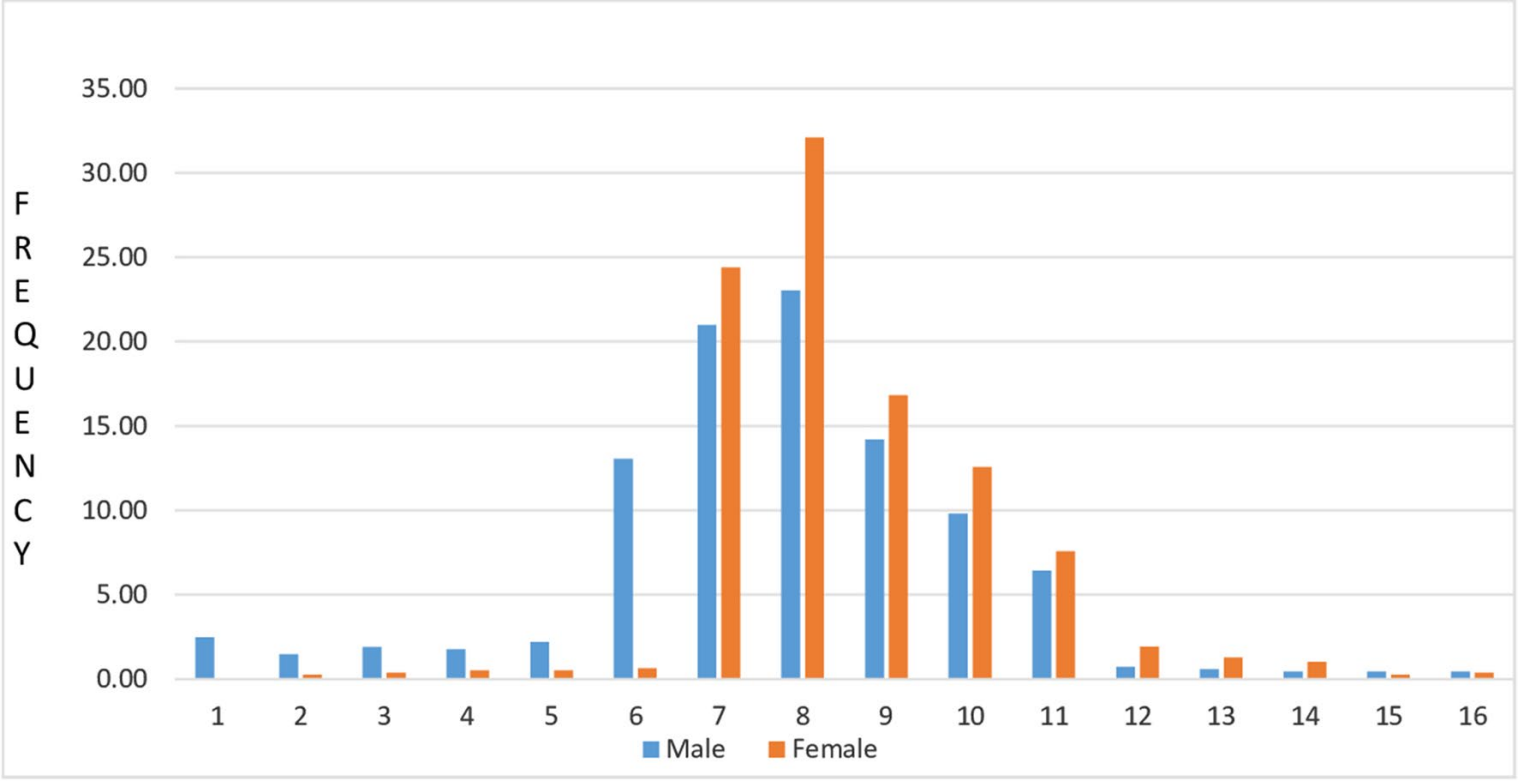
Histogram of G6PD activity distribution by sex

The overall prevalence of G6PD deficiency was observed to be 3.2% (n = 47) with an average erythrocytic enzyme activity of 1.26 ± 0.65 IU/g Hb in males and 3.8 ± 1.8 IU/g Hb in females, while the average enzyme activity for all the individuals was 7.4 ± 0.8 IU/g Hb. Out of 13 PVTGs, G6PD deficiency was found to be prevalent among nine tribes (Bonda, Didayi, Dongria Kondha, Chuktia Bhunjia, Hill Kharia, Kutia Kondha, Lanjia Saora, Paudi Bhuyan, and Saora), the lowest in Didayi (1.1%) and the highest in Hill Kharia (10.4%). Gender-wise G6PD activity distribution of the entire population is shown in Fig. 2. Molecular analysis of the G6PD gene identified mutations corresponding to the G6PD Orissa and Mediterranean variants in 97.9% and 2.1% of the cases respectively (Table 5). The allele frequency of the G6PD Orissa variant was 0.035 and 0.015 in male and female populations, respectively. The G6PD Mediterranean variant was observed only in male population with an allele frequency of 0.002.

**Table 5:** Prevalence of G6PD deficiency and malaria among the 13 PVTGs of Odisha

PVTG Tribes	Total Study Population	G6PD deficiency prevalence	G6PD variant	G6PD activity in male deficient cases (IU/g Hb)	G6PD activity in Female deficient cases (IU/g Hb	G6PD activity (IU/g Hb)	Malaria Distribution				Malaria positive in G6PD deficient cases			
							Pf	Pv	Pf + Pv	Total	Pf	Pv	Pf + Pv	Total
Bonda	77	3 (3.9%)	Orissa	1.1 ± 0.34	3.3	1.1 ± 0.34	3 (50%)	3 (50%)	ND	6 (7.8%)	ND	3 (100%)	ND	3(100%)
Chuktia Bhunjia	59	3 (5.1%)	Orissa	1.2 ± 0.45	3.6	1.2 ± 0.45	ND	ND	3	3 (5.1%)	ND	ND	3 (100%)	3(100%)
Didayi	84	1 (1.1%)	Mediterranean	0.9	NA	0.9	1 (50%)	1 (50%)	ND	2 (2.4%)	ND	1	ND	1(100%)
Dongoria Kondha	145	7 (4.8%)	Orissa	1.3 ± 1.20	4.5 ± 0.6	1.3 ± 1.20	14 (70%)	2 (10%)	4 (20%)	20 (13.8%)	2 (40%)	2 (40%)	1 (20%)	5 (71.4%)
Hill Kharia	48	5 (10.4%)	Orissa	1.2 ± 0.86	3.7 ± 0.3	1.2 ± 0.86	ND	2 (66.7%)	1 (33.3%)	3 (6.3%)	ND	1 (50%)	1 (50%)	2 (40%)
Mankidia /Birhor	29	ND	ND	ND	ND	ND	ND	ND	ND	ND	ND	ND	ND	ND
Juanga	109	ND	ND	ND	ND	ND	ND	ND	ND	ND	ND	ND	ND	ND
KutiaKondho	152	2 (1.3%)	Orissa	1.3 ± 0.07	ND	1.3 ± 0.07	29 (76.3%)	3 (7.9%)	6 (15.8%)	38 (25%)	1 (50%)	1 (50%)	ND	2(100%)
Lodha	102	ND	ND	NA	ND	NA	ND	ND	ND	ND	ND	ND	ND	ND
LanjiaSaora	148	8 (5.4%)	Orissa	1.4 ± 0.09	3.8 ± 1.6	1.4 ± 0.09	ND	3 (100%)	ND	3 (2%)	ND	3 (100%)	ND	3 (37.5%)
PaudiBhyan	309	4 (1.3%)	Orissa	1.3 ± 0.54	3.4 ± 1.2	1.3 ± 0.54	24 (70.6%)	6 (17.6%)	4 (11.8%)	34 (11%)	1 (25%)	3 (75%)	ND	4(100%)
Saora	199	7 (3.5%)	Orissa	1.2 ± 0.64	4.4 ± 1.4	1.2 ± 0.64	3 (33.3%)	4 (44.4%)	2 (22.2%)	9 (4.5%)	2 (50%)	1 (25%)	1 (25%)	4 (57.1%)
Total	1461	47 (3.2%)	NA	1.2 ± 0.65	3.8 ± 1.8	1.2 ± 0.65	74 (62.7%)	24 (20.3%)	20 (16.9%)	118 (8.1%)	6 (22.2%)	15 (55.5%)	6 (22.2%)	27 (57.4%)

*PVTGs* Particularly Vulnerable Tribal Groups; *G6PD* glucose 6-phosphate dehydrogenase; *Pf* *Plasmodium falciparum;* *Pv* *Plasmodium vivax*; *Pf + PV* *P.falciparum* and *P. vivax*; *ND* not detected; *NA* not applicable

### Haemoglobin status among sickle cell, thalassaemia, and G6PD deficiency individuals

The average haemoglobin (Hb) level of the individuals having HbAS with α deletion was 12.2 g/dl (range: 10.8–13.1) whereas HbAS individuals without α gene deletion have 11.3 g/dl (range: 9.00–10.6), β-thal have 11.02 g/dl (range: 8.6–12.6) and the HbS/β-thal compound heterozygote individuals have 10 g/dl. The average haemoglobin in HbAS male and female was 10.8 ± 2.3 and 11.7 ± 2.1, respectively. The individuals with G6PD deficiency had significantly (P = 0.023) low levels of Hb (Male: 9.5 ± 0.7 g/dl, Female: 8.9 ± 1. 1 g/dl) than the normal individuals (Male: 11.7 ± 2 g/dl, Female: 10.8 ± 1.7 g/dl). Further age-wise stratification of G6PD female individuals, the Hb level of female in reproductive age group (8.8 ± 0.5 g/dl) was found to be significantly lower compared to the females of other age group (≤ 14 years.: 10.1 ± 0.8 g/dl, ≥ 50 years: 8.8 ± 1.1 g/dl) as shown in Table 6.

**Table 6:** Mean Haemoglobin level in Sickle cell, β-Thalassaemia, G6PD deficiency and Normal-controls of the studied PVTGs

Age	β-thalassaemia + Sickle cell anaemia (n = 56)		G6PD deficiency (n = 47)		Normal control (n = 1017)	
	Male Hb (g/dl)	Female Hb (g/dl)	Male Hb (g/dl)	Female Hb (g/dl)	Male Hb (g/dl)	Female Hb (g/dl)
≤ 14 years	11.8 ± 1.2 (n = 6)	10.6 ± 0.8 (n = 3)	10.3 ± 0.6 (n = 7)	10.1 ± 0.8 (n = 2)	11.0 ± 1.7 (n = 148)	10.6 ± 1.6 (n = 112)
15–49 years	11.5 ± 1.2 (n = 8)	12.1 ± 1.3 (n = 16)	9.4 ± 0.5 (n = 12)	8.8 ± 0.5 (n = 11)	12.4 ± 2.1 (n = 199)	11.0 ± 1.7 (n = 296)
≥ 50 years	11.0 ± 1.3 (n = 11)	11.3 ± 1.3 (n = 12)	9.2 ± 0.7 (n = 8)	8.8 ± 1.5 (n = 7)	11.6 ± 2.0 (n = 133)	10.7 ± 1.9 (n = 129)
All	10.8 ± 2.3 (n = 25)	11.7 ± 2.1 (n = 31)	9.5 ± 0.7 (n = 27)	8.9 ± 1. 1 (n = 20)	11.7 ± 2.0 (n = 480)	10.8 ± 1.7 (n = 537)

### Malaria

Of the 1461 individuals enrolled in study, 118 (8.1%) individuals were diagnosed to be positive for malaria. Amongst the malaria positives, 62 were due to *P. falciparum* and 24 (20.3%) were due to *P. vivax*, while 20 (16.9%) were mixed infections (*P falciparum* and *P. vivax*). The prevalence of malaria was high in Kutia Kondho, Dongoria Kondho, and Paudi Bhuyan. Out of 50 sickle cell individuals (48 HbAS and 2 HbSS), 8 (16%) were found to be infected with malaria (Table 2). Of the total G6PD deficiency individuals 57.4% (27 out of 47) were found to be malaria positive. In Bonda, Chutkiya Bhunjia, Didayi, Kutia Konda and Paudi Bhuyan, all the G6PD deficient individuals were malaria positive as well (Table 5).

## Discussion

The PVTGs are the marginalized section of the scheduled tribes of India, who are relatively isolated, educationally and socio-economically backward, living in habitats far away from amenities and having stagnant or declining populations. Among the 75 enlisted PVTGs by the Government of India, the highest number are found in Odisha (13) with a population of around 0.25 million, who are residing in 12 districts (*Mayurbhanj, Keonjhar, Deogarh, Anugul, Sundargarh, Kandhamal, Rayagada, Malkangiri, Kalahandi, Gajapati, Ganjam and Nuapada*) [29]. This report represents the first detailed population survey assessing the frequencies of classical erythrocyte genetic disorders along with malaria in 13 PVTGs (*Juang, Bondo, Didayi, Langia Saora, Saora, Kutia Kondho, Dongoria Kondho, Paudi Bhuyan, Birhor, Chuktia Bhunjia, Kharia, Lodha, Mankidia*) found in Odisha. The study shows an overall allele frequency of the HbS gene as 0.019, β-thal as 0.0009, α-thal as 0.0113, and G6PD deficiency as 0.022 among the studied PVTGs, while the malaria test positive rate (TPR) was 8.1. The most important finding in the present study was α-thal gene among certain PVTGs not reported earlier.

Since the first report of sickle cell disease by Lehman and Cutbush [30] among the tribal populations in Nilgiri Hills in South India, many clusters of the tribal populations have been screened and the frequency of the sickle cell mutation and other abnormal haemoglobins have been recorded [31]. According to the latest estimate around 12,000 infants are born every year with clinically significant haemoglobinopathies, of which sickle-cell disorders and β-thalassaemia major account for almost equal numbers [32]. In Odisha, studies have shown the prevalence of sickle cell disease (< 5–15%), β-thalassaemia (6.3% to 8.5%) and α-thalassaemia (34.5% to − 56.2%) in different tribal groups [6, 33–35], while the prevalence of sickle cell in certain PVTGs was 1.3% to 10. 1% (Juang: 1.3%, Bondo: 1.6%, Saora: 3.0%, Didayi: 8.7% and Kutia Kondh: 10.1%) [36–39]. The present study is an expansion to all 13 PVTGs, where β^S^ gene carrier (3.4%) was found in eight “primitive tribes” (Juang: 3.6%, Bondo: 2.6%, Didayi: 7.1%, Langia Saora: 5%, Saora: 5.5%, Kutia Kondho: 3.3%, Dongoria Kondho: 2.0%, and Paudi Bhuyan: 3.2%), β^thal^ (0.3%) and β^S^-β^thal^ compound heterozygote gene (0.07%) in one “primitive tribes” (Paudi Bhuyan) and α^thal^ and β^S^ gene co-inheritance in around 2.1% of the individuals in five (Didayi, Dongoria Kondho, Paudi Bhuyan, Lagia Saora, and Saora) “primitive tribes”. The Hardy–Weinberg-Equilibrium (HWE) analysis calculated using the gene frequency of unrelated individuals shows genetic equilibrium in all PVTGs except in the Paudi Bhuyan tribe for β^S^ and in Saora for α^thal^ indicating the influence of genetic drift or genetic migration among Paudi Bhuyan and Saora tribes, whereas others are still isolated and maintain genetic homogeneity. DNA analysis has revealed that the majority (~ 56%) of α^thal^ mutations in the study population is due to either αα/α-3.7 or αα/α-4.2 gene deletion similar to the observations made in other Indian tribes [40, 41]. Whereas molecular analysis has shown that the chromosomes bearing the β^S^ gene are linked to Arab-Asian haplotype and β^−thal^ gene with IVS 1–5 mutations suggesting unicentric origin of the mutation in PVTGs of Odisha as predicted for other tribes in India [42].

In spite of a higher carrier frequency of sickle cell mutation, detection of a low percentage of the sickle-cell homozygote (0.1%) cases in the studied PVTG population could be due to increased mortality during infancy and early childhood because of poor nutritional/haemoglobin status and malaria infection [43]. As per the NVBDCP-Odisha malaria data the Annual Parasite Index (API) was quite high in these studied districts (Mayurbhanj: 10.9, Keonjhar: 14.6, Deograh: 14.3, Anugul: 22.3, Sundargarh: 12.4 Kandhamal: 47.5, Rayagada*:* 65.3, Malkangiri: 44.8 Kalahandi: 20.3, Gajapati: 18.3, Ganjam: 4.2 and Nuapada: 8.7) suggesting high transmission [44]. However, diagnostic tools for haemoglobinopathies in primary health system are lacking in Odisha. Hence, the health-system diagnostic capacities need to be strengthened to optimize the investigation of genetic haemoglobin disorders and their consequences. Further, co-inheritance of α^thal^ and β^S^ gene among some of the PVTGs is very significant, because α^thal^ can reduce the amount of sickled RBC, increase the HbF/HbA2 level and reduce haemolysis eventually diminishing the severity of the disease [45]. Hence co-evolution of α^thal^ and β^S^ heterozygotes, in this group of the population could be clinically advantageous and might be giving protection against *P. falciparum*. An intensive study is therefore, required in this setting to generalize the data to African populations in this regard [46].

G6PD deficiency plays a central role in malaria elimination programme that use PQ as part of the treatment of choice for both *P. falciparum* and *P. vivax*. However, the risk of haemolysis in G6PD deficient individuals hampers the widespread use of PQ safely in malaria-infected patients. There has been a sharp decline in malaria cases in Odisha [47] since 2018.If this trend continues, the state will enter a pre-elimination phase soon, during which it will be essential to screen for asymptomatic malaria in addition to G6PD deficiency. The present study has revealed a moderate to high prevalence (1.1–10.4%) of G6PD deficiency in nine out of 13 PVTGs (Bonda, Didayi, Hill Kharia, Lanjia Saora, Soura, Paudi Bhuyan, Chuktia Bhunjia, Dongria Kondha, Kutia Kondha) and 57.4% of the G6PD deficiency individuals were found to be positive for malaria (*P. falciparum*: 12.7%, *P*. *vivax*: 32.6%, *P. falciparum* + *P. vivax*: 12.76%) indicating high transmission. Since G6PD deficiency is very common in malaria-endemic areas, WHO have recommended G6PD testing before giving PQ [48]. G6PD testing is rarely done in Odisha. Similar to other tribes of Odisha and India, G6PD genotype analysis confirmed that almost all deficiency amongst the PVTGs was caused by G6PD Orissa (131C → G) followed by G6PD Mediterranean (563C → T) [20, 49]. The WHO, groups, the G6PD Orissa variant into a kind of “moderately deficient” (Class III) associated with symptomatic haemolysis on exposure to certain drugs and infectious agents, and G6PD Mediterranean “severely deficient” (Class II) associated with drugs induced haemolytic anaemia (DIHA), favism and neonatal jaundice. The present study has detected a significantly low level of Hb among the G6PD deficiency individuals compared to the individuals with normal enzyme activity. More importantly, the Hb level among the G6PD deficiency individuals infected with malaria had significantly low (p < 0.05) Hb level (8.9 ± 1.1 g/dl) compared to their counterparts without malaria infection (9.4 ± 0.5 g/dl) indicating malaria infection triggering the haemolysis. Therefore, G6PD testing among the PVTGs of this region is strongly recommended before treating with PQ for *P. falciparum* and *P vivax* malaria.

In conclusion, a high frequency of alleles such as G6PD deficiency, HbS, and α-thalassaemia associated with malaria are prevalent among the PVTGs of Odisha. Moderate to high frequency of G6PD deficiency indicates that the diagnosis and management of G6PD deficiency are necessary and an obligatory new-born screening programme, prenatal screening, and counselling for these genetic disorders, especially HbS, are required for the PVTGs. The most significant challenge in delivering the health services in PVTG areas is the poor physical access due to non-availability of health care service providers. In such a case, Mobile Health Units (MHU) at least once a week might be considered as an alternative option to address such health issues in PVTG-specific areas of Odisha.

## Data Availability

The data generated or analysed during the current study are available from the corresponding author on reasonable request.
